# Direct oral anticoagulants vs vitamin K antagonist on dementia risk in atrial fibrillation: systematic review with meta-analysis

**DOI:** 10.1007/s11239-023-02843-5

**Published:** 2023-07-05

**Authors:** Diogo R. Branco, Mariana Alves, Catarina Severiano E Sousa, João Costa, Joaquim J. Ferreira, Daniel Caldeira

**Affiliations:** 1 Faculdade de Medicina, Universidade de Lisboa, Lisbon, Portugal; 2 Serviço de Medicina III, Hospital Pulido Valente, CHLN, Lisbon, Portugal; 3 Laboratory of Clinical Pharmacology and Therapeutics, Faculdade de Medicina, Universidade de Lisboa, Av. Prof. Egas Moniz, 1649-028 Lisbon, Portugal; 4 Faculdade de Medicina, Instituto de Medicina Molecular, Universidade de Lisboa, Lisbon, Portugal; 5 CNS-Campus Neurológico Senior, Torres Vedras, Portugal; 6 Centro Cardiovascular da Universidade de Lisboa-(CCUL@RISE), CAML, Faculdade de Medicina, Universidade de Lisboa, Lisbon, Portugal; 7 Serviço de Cardiologia, Hospital Universitário de Santa Maria-CHULN, Lisbon, Portugal; 8 Centro de Estudos de Medicina Baseada na Evidência (CEMBE), Faculdade de Medicina, Universidade de Lisboa, Lisbon, Portugal

**Keywords:** Anticoagulation, DOAC, VKA, Dementia, Atrial fibrillation

## Abstract

Oral anticoagulation significantly reduces the incidence of dementia in atrial fibrillation patients. However, this protective effect has not been compared between Direct Oral Anticoagulants (DOAC) and Vitamin K antagonists’ anticoagulants (VKA). We conducted an electronic search for potentially eligible studies through the bibliographic databases MEDLINE, CENTRAL, ClinicalTrials.gov, EMBASE and Web of Science. The outcome of interest was dementia. Random-effects meta-analysis was performed. Nine observational studies were included and 1,175,609 atrial fibrillation patients were enrolled. DOAC therapy was associated with a significant reduction when compared with patients under VKA therapy (hazard ratio 0.89; 95% confidence interval 0.80–0.99). The grade of confidence of our results was very low due to the risk of bias. DOAC therapy is associated with a significant decrease in the risk of dementia when compared with VKA therapy. However, the low certainty of the evidence along with the paucityof clinical trials dedicated to answering this important question underscores a need for global clinical research initiatives.

**Supplementary Information:**

The online version contains supplementary material available at 10.1007/s11239-023-02843-5.

## Introduction

Atrial fibrillation (AF) is the most prevalent cardiac arrhythmia, affecting more than 33 million people worldwide, the majority of which are older people [1, 2]. Likewise, neurocognitive impairment and dementia are very common in this age group, affecting more than 50 million people, which corresponds to 5–8% of > 60 years old population at any given time [3], both of which share numerous risk factors with atrial fibrillation, such as older age, hypertension, sleep apnea, diabetes mellitus, vascular disease, heart failure and alcohol consumption [4].

Several recent studies demonstrated an association between AF and increase risk of cognitive decline [5, 6] and faster decline within 7 years from the development of the mentioned cardiac arrhythmia [7], both in patients with and without history of stroke [8] and particularly in older people aged < 70 years old [9]. AF is an independent risk factor for any subtype of dementia (senile, vascular, Alzheimer’s and non-specified dementia) [9, 10].

It is conceivable that by preventing future embolic events with effective oral anticoagulation, this therapy would be effective in preventing dementia in AF patients. Several systematic reviews concluded that oral anticoagulation reduced significantly the incidence of cognitive impairment and dementia in AF patients [11, 12], probably due to the reduction of ischemic cerebrovascular events in AF patients, both with and without clinical repercussions [13–15].

However, there is no clear evidence on which oral anticoagulant therapy is better at preventing dementia in AF patients, direct oral anticoagulation (DOAC) or vitamin K antagonist anticoagulation (VKA). Therefore, the purpose of this systematic review is to compare DOACs and VKA regarding dementia risk in patients with atrial fibrillation.

## Methods

This systematic review was conducted using PRISMA [16] and MOOSE guidelines [17]. The protocol was registered in PROSPERO: CRD42020215699.

### Eligibility criteria

For this systematic review, we considered the published randomised controlled trials and observational longitudinal controlled studies which evaluated AF-diagnosed patients, defined as a supraventricular tachyarrhythmia with uncoordinated atrial electrical activation and consequently ineffective atrial contraction [18], abnormal electrocardiogram (ECG) activity and compatible clinical criteria (previous diagnosis made by the patient’s physician, or corresponding administrative code were also acceptable in the definition of the patient’s condition), treated with DOACs (also named NOACs), such as dabigatran, apixaban, edoxaban or rivaroxaban, in comparison with VKA, such as warfarin, phenprocoumon and acenocoumarol. Studies were considered for inclusion irrespective of baseline posology, study follow-up, funding and language of publication.

### Information sources and search method

The search was performed from its inception date to September 2021 and potentially eligible studies were identified through an electronic search in the bibliographic databases MEDLINE, Cochrane Central Register of Controlled Trials (CENTRAL), ClinicalTrials.gov, EMBASE and Web of Science. Reference lists of systematic reviews, as well as the reference list of included studies, were comprehensively searched. The search strategy, including terms used for the database search, are available in Supplementary table 1.

### Study selection and outcome measures

After excluding duplicate records obtained in the electronic search, studies were included if they: (1) were RCTs or observational studies, (2) included AF patients, (3) assessed dementia, (4) compared DOAC and VKA, (5) had no previous diagnosis of dementia. All studies that had a cross-sectional design, did not present original data, had incomplete outcomes, had no comparators or were expert opinions, editorials, case reports, case series or systematic reviews, were excluded.

The primary outcome of interest was the incidence of dementia in patients with atrial fibrillation, under DOAC or VKA, defined as a decline from the previous levels of cognitive functioning and performing, corresponding to impairment in two or more cognitive domains (attention, executive function, memory, language, visuospatial function), which interfere with the ability to function at work or usual activities and is not better explained by delirium or major psychiatric disorder (dementia NIA-AA criteria) [19]. For the evaluation of this outcome, we didn't restrict the diagnosis criteria used: we accepted Mini-Mental State Examination (MMSE) cut-offs, International Classification of Diseases (ICD) 9/ICD 10 dementia criteria, Diagnostic and Statistical Manual of Mental Disorders (DSM) IV/V dementia criteria, National Institute on Aging—Alzheimer’s Association workgroups on diagnostic guidelines for Alzheimer’s disease (NIA-AA) dementia criteria and comprehensive neuropsychological assessment. The clinical judgement criteria made by the patient's physician/researcher and administrative codes were also acceptable.

### Studies records and data extraction

The records retrieved through electronic database search were screened independently by two authors (DB, MA). Suitable studies were evaluated for inclusion in the review through full-text assessment. Study selection and data extraction were performed independently. If different data were available for the same trial, the most recent report was considered. We also contacted authors when data was missing, such as primary outcome data and study characteristics. If the authors did not respond, the study was excluded.

Two reviewers (DB, MA) independently extracted data from the included observational studies using a standardised electronic form. Disagreements were resolved by consensus or with the help of a third author (DC). Study characteristics and results were extracted independently into a standardized form.

When only a composite outcome with dementia included was reported, we include it in the analysis. If only odds ratio (OR) was reported, we converted it to relative risk (RR) and assumed it to be similar to hazard ratio (HR) [20]. If results from multiple multivariable models were presented, we extracted associations from the most fully adjusted model.

### Data evaluation, synthesis and analysis

The ROBINS-I tool was used to assess the risk of bias in observational studies [21]. The seven predefined specific domains of analysis were: confounding, selection of participants into the study, classification of interventions, deviations from intended interventions, missing data, measurement of outcomes and selection of the reported result. Two independent review authors (DB and MA) performed critical assessments for each domain of the risk of bias tool. Disagreements throughout this process were resolved by consensus or through a third author (DC).

The outcome was treated as dichotomous data. We used the adjusted data whenever available. The data was pooled using RevMan version 5.3.3 (The Nordic Cochrane Centre, Copenhagen; The Cochrane Collaboration, 2014) and STATA 17.0 and meta-analyses were performed using the random effects method weighted by the inverse variance to estimate pooled HR and 95% confidence interval (95% CI). Heterogeneity was assessed using the Chi^2^ test (threshold P > 0.10) and through I^2^ statistics, considering statistical heterogeneity as low if I^2^ < 25%; moderate if I^2^ 25–75%; and high if I^2^ > 75%. The I^2^ statistics measures the percentage of total variation between studies attributed to interstudy heterogeneity rather than random heterogeneity [22]. Publication bias assessment was performed through funnel plot examination if more than 10 studies were included [23, 24].

Subgroup analyses on the primary outcome were carried out regarding (a) the duration of follow-up (FU) time, considering long FU when the follow-up period was ≥ 5 years and short FU when it was < 5 years. The cut-off value was defined as 5 years since current evidence suggests that the overall risk of dementia in AF patients appears to be higher in studies with more than 5 years of follow-up [10, 25]; (b) risk of bias (high vs moderate risk of bias), since studies with a higher risk of bias could camouflage or overestimate the effect of an intervention; (c) single/composite outcome reported since the composite outcomes introduce additional data that are not of interest for the goal of this study.

As recommended by the Grading of Recommendations Assessment, Development and Evaluation (GRADE) Working Group methodology, two reviewers independently (DB and MA) assessed the outcome in the following domains: risk of bias, inconsistency, indirectness, imprecision, and publication bias [26, 27]. The confidence in the pooled evidence was graded as very low, low, moderate, or high. The pooled hazard risks, as well as the confidence in the pooled evidence, were reported in Supplementary table 2.

## Results

### Study selection

The search of electronic databases yielded 607 published studies. After title and abstract screening, 23 studies were selected for full-text assessment, of which 14 were rejected (Supplementary Fig. 1). Of the remaining nine studies, all were retrospective cohort studies [28–36].

Overall, the studies included 1,175,609 AF patients, with a median age ranging from 65.9 to 86.1 years old and a median follow-up period ranging from 243 days to 9 years. Six of the included studies [29–31, 33, 34, 36] had a shorter follow-up period than 5 years from the beginning of the studies.

Overall, five studies assessed incidence of dementia using ICD-9/ICD-10 [29–31, 33, 34], one using MMSE cut-offs [35] and the remaining three using non-specified administrative code/physician’s diagnosis [28, 32, 36].

Regarding the time in therapeutic range (TTR) in patients under Warfarin treatment, three studies stated mean values above 65% [28, 29, 33], and the remaining studies did not stated any value regarding this subject.

### Study characteristics

Main study characteristics, including study design, patient demographics and clinical characteristics, are reported in Table 1. In general, characteristics were well balanced between both study groups. The majority of AF patients included were males with more than 65 years of age. There were also comorbidities associated with patients from both groups, such as cardiovascular ones, but the specific comorbidity prevalence was highly variable between studies, as reported in Table 2.

**Table 1:** Studies characteristics of the included studies

Study year	Design	Region	Study population	Mean age	% female	Intervention	Comparator	Mean follow up	Outcome	Outcome assessment
Chen et al. 2018	Retrospective cohort study	USA	(MARKETSCAN) 307 099 patients with nonvalvular AF; (optum) 161 346 patients with nonvalvular AF	(MarketScan) 67 years; (optum) 73 years	(MarketScan) 5%; (optum) 45%	DOAC (89,811 patiens in MarketScan; 49,402 patients on optum)	VKA (89,811 patiens in MarketScan; 49,402 patients on optum)	0.7–2.2 years	Dementia	ICD-9/ICD-10
Friberg et al. 2019	Restrospective cohort study	Sweden	444,106 AF patients	(With OAC at baseline) 73.7 years; (without OAC at baseline) 75.7 years	(With OAC at baseline) 40.6%; (without OAC at baseline) 48.1%	DOAC (12,916 patients)	VKA (190,769 patients)	9 years	Dementia	ICD-9/ICD-10
Jacobs et al. 2016	Restrospective cohort study	USA	5254 patients	72.4 ± 10.9 years	41.0%	DOAC (2627 patients)	VKA (2627 patients)	243 days	Composite outcome of dementia, stroke, and TIA	NR
Kim et al. 2020	Restrospective cohort study	South Korea	53,236 OAC-naive AF patients	DOAC: 73 years; warfarin: 70 years	DOAC: 43.2%; warfarin: 39.1%	DOAC (28,683 patients)	VKA (24,553 patients)	20.2 months	Dementia	ICD-9/ICD-10
Mongkhon et al. 2020	Restrospective cohort study	UK	84,521 AF patients	NR	NR	DOAC (4657 patients)	VKA (12,880 patients)	5.9 years	Composite of new-onset dementia/cognitive impairment	NR
Sogaard et al. 2019	Restrospective cohort study	Denmark	34,683 incident OAC users with hospital-diagnosed AF	[60–69 yo] warfarin 65.9 (2.7); DOAC 65.9 (2.7) [70–79 yo] warfarin 74.9 (2.8); DOAC 74.7 (2.9) [80–89 yo] warfarin 85.1 (3.8); DOAC 86.1 (4.4)	[60–69 yo] 37.0%; [70–79 yo] 45.7% [80–89 yo] 59.8%	DOAC (21,311 patients)	VKA (13,372 patients)	3.4 years	Dementia	ICD-9/ICD-10
Hsu et al. 2021	Retrospective cohort study	Taiwan	12,068 AF patients	NR	DOAC: 2442 (40.5%) VKA: 2474 (41.0%)	DOAC (6034 patients)	VKA (6034 patients)	DOAC: 3.27 years VKA: 3.08 years	Dementia	ICD-9/ICD-10
Kundnani et al. 2021	Retrospective cohort study	Romania	450 AF patients	NR	DOAC: 163 (56.8%) VKA: 84 (51.5%)	DOAC, apixaban (287 patients)	VKA, acenocoumarol (163 patients)	5 years	Dementia	MMSE
Lee et al. 2021	Retrospective cohort study	South Korea	72,846 AF patients	71.8 ± 10.5 years	42.0%	DOAC (46 898 patients)	VKA (25 948 patients)	4 years	Dementia	NR

*AF* atrial fibrillation, *A* apixaban, *D* dabigatran, *R* rivaroxaban, *DOAC* direct oral anticoagulation, *NR* not reported, *VKA* Vitamin K antagonist, *yo* years old

**Table 2:** Risk factors of the included studies’ patients

Study year	Number of patients	Age (years)		﻿Men		Hypertension		﻿Diabetes		﻿Dyslipidaemia		﻿Coronary artery disease/isquemic heart disease		﻿Heart failure		﻿Stroke/transient ischemic attack	
		DOAC	VKA	DOAC	VKA	DOAC	VKA	DOAC	VKA	DOAC	VKA	DOAC	VKA	DOAC	VKA	DOAC	VKA
Chen et al. 2018																	
MarketScan	DOAC: 90,012 VKA:217,087	D: 67 R: 67 A: 69	70	D: 65% R: 62% A: 60%	D:75% R: 75% A: 80%	D:75% R: 75% A: 80%	70%	D: 29% R: 28% A: 30%	30%	D: 57% R: 55% A: 59% (*)	57% (*)	D: 7.9% R: 8.2% A: 9.3%	21%	D: 25% R: 24% A: 28%	29%	D: 19% R: 17% A: 20%	21%
Optum	DOAC: 49,451 VKA: 112,051	D: 69 R: 70 A: 73	73	D: 63% R: 60% A: 55%	D: 85% R: 85% A: 88%	D: 85% R: 85% A: 88%	84%	D: 34% R: 35% A: 37%	37%	D: 59% R: 60% A: 64% (*)	59% (*)	D: 11% R: 12% A: 14%	14%	D: 30% R: 31% A: 36%	38%	D: 23% R: 25% A: 29%	26%
Friberg et al. 2019	202,946	73.7		59.4%		53.2%		53.2%		37.9% (*)		16.5%		31%		21.9%	
Jacobs et al. 2016	DOAC: 2627 VKA: 2627	﻿71.2 ± 11.9	﻿73.5 ± 9.6	﻿59.6%	58.4%	﻿76.5%	﻿80.0%	﻿29.5%	﻿31.4%	64.6%	﻿60.9%	﻿﻿39.7%	﻿41.7%	﻿﻿30.5%	﻿22.7%	﻿10.8%	﻿10.7%
Kim et al. 2020	DOAC: 28,683 VKA: 24,553	73 (66–78)	70 (62–77)	16,290 (56.8)	14,958 (60.9)	24,767 (86.3)	19,385 (79.0)	8935 (31.2)	7734 (31.5)	25,922 (90.4)	20,959 (85.4)	3511 (12.2)	2977 (12.1)	16,892 (58.9)	13,493 (55.0)	13,340	10,240
Mongkhon et al. 2020	DOAC: 4657 VKA: 12,880	74.3 (10.3)	74.4 (9.7)	2576 (55.3%)	7187 (55.8%)	159 (3.4%)	460 (3.6%)	136 (2.9)	338 (2.6)	37 (0.8)	131 (1.0)	231 (5.0)	755 (5.9)	417 (9.0)	1200 (9.3)	496 (10.7)	1154 (9.0)
Sogaard et al. 2019																	
60–69 yo	DOAC: 6846 VKA: 4332	65.9 (2.7)	65.9 (2.7)	62.2%	58.3 (3992)	58.3 (3992)	60.7 (2629)	11.1 (761)	14.2 (615)	NR	NR	8.0 (550)	11.0 (475)	11.2 (765)	16.9 (731)	NR	NR
70–79 yo	DOAC: 8126 VKA: 5387	74.7 (2.9)	74.9 (2.8)	53.1%	62.2 (5057)	62.2 (5057)	65.0 (3504)	11.6 (940)	13.6 (731)	NR	NR	10.2 (829)	15.0 (809)	15.5 (1263)	21.1 (1136)	NR	NR
≥ 80 yo	DOAC: 6339 VKA: 3653	86.1 (4.4)	85.1 (3.8)	37.9%	65.0 (4119)	65.0 (4119)	69.1 (2525)	10.8 (686)	11.6 (422)	NR	NR	13.0 (825)	17.1 (625)	28.0 (1774)	34.2 (1250)	NR	NR
Hsu et al. 2021	DOAC: 17,065 VKA: 8024	< 65 yo: 2878 (16.9) 65–74 yo: 5558 (32.6) > 75 yo: 8629 (50.6)	< 65 yo: 2998 (37.4) 65–74 yo: 2146 (26.7) > 75 yo: 2880 (35.9)	9653 (56.6%)	4781 (59.6)	14,019 (82.2)	6464 (80.6)	6205 (36.4)	3119 (38.9)	6337 (37.1) (*)	2445 (30.5) (*)	7748 (45.4)	3923 (48.9)	5129 (30.1)	3075 (38.3)	6952	3081
Kundnani et al. 2021	DOAC: 287 VKA: 163	NR	NR	43.2%	48,5%	NR	NR	NR	NR	NR	NR	NR	NR	NR	NR	NR	NR
Lee et al. 2021	DOAC: 46,898 VKA: 25,948	72.7 ± 9.9 yo	70.1 ± 11.2 yo	56.5%	59.5	85.8%	81.1%	27.1%	25.4%	56.7%	51.1%	5.8%	6.0%	45.6%	40.8%	25.0%	27.0%

*A* apixaban, *D* dabigatran, *R* rivaroxaban, *DOAC* direct oral anticoagulation, *NR* not reported, *VKA* vitamin K antagonist, *yo* years old; (*) lipid lowering drugs

### Risk of bias

According to the ROBINS-I tool, all studies were classified as moderate overall risk of bias, except for [28, 32, 36] which were considered as serious risk due to the unspecified method of dementia outcome assessment.

The risk of bias classification for each study regarding the primary outcome can be consulted in Supplementary table 3.

### Primary outcome: dementia

**Fig. 1:**
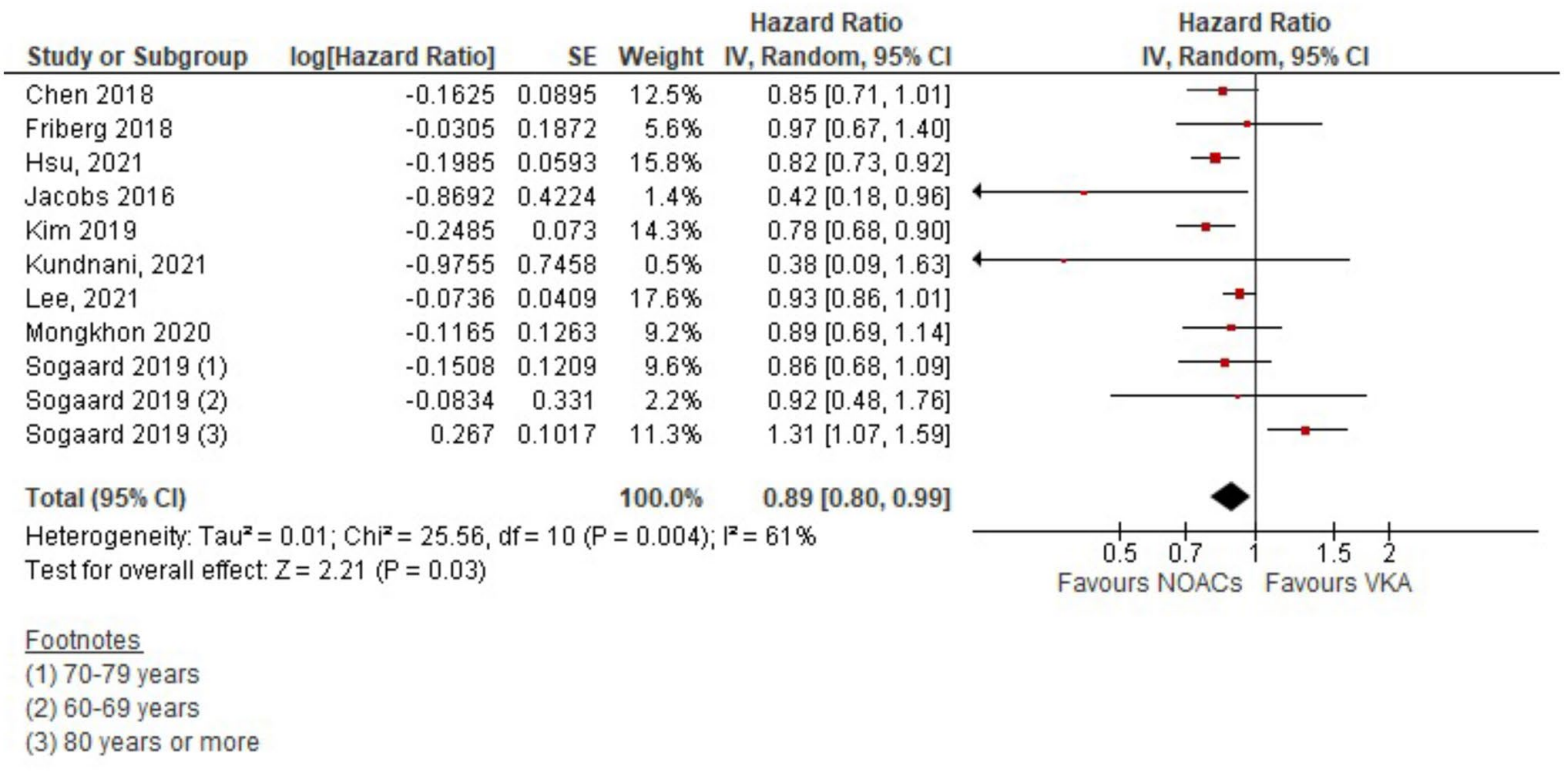
Hazard ratio for dementia in patients with atrial fibrillation according to anticoagulant (DOAC vs VKA)

Adjusted pooled results showed that the risk of dementia outcomes in patients under DOAC therapy was associated with a significant reduction (HR 0.89; 95% CI 0.80, 0.99) when compared with patients under VKA therapy (Fig. 1). There was moderate statistical heterogeneity (I^2^ = 61%; P = 0.004).

### Subgroup analysis

**Fig. 2:**
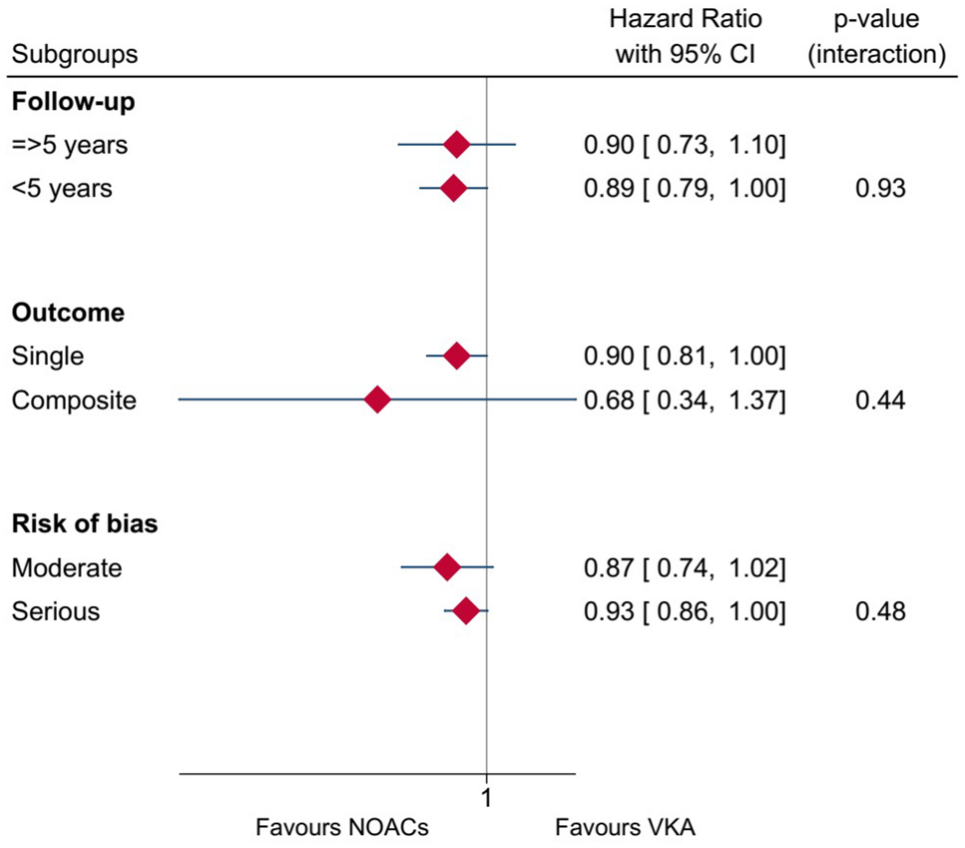
Hazard ratio for risk of bias subgroup analysis (moderate vs serious risk), follow-up period subgroup analysis (short vs long follow-up) and outcome subgroup analysis (single vs composite outcome)

Figure 2 presents subgroup analyses for the duration of follow-up time (long vs short FU), risk of bias (high vs moderate risk of bias) and composite outcome. There was no statistically significant difference between the risk of bias and follow-up period subgroups. Two studies reported composite outcomes including stroke and TIA besides dementia [29] and new-onset dementia and cognitive impairment [32]. In the composite outcome subgroup, the DOAC’s effect was not statistically significant in reducing dementia risk (HR 0.68, 95% CI 0.34, 1.38; I^2^ = 66%). Additionally, the overall statistically significant difference did not persist in the remaining seven studies after the exclusion of the composite outcome subgroup (HR 0.90, 95% CI 0.81, 1.00; I^2^ = 64%).

The subgroup analyses with the data of each study can be consulted in Supplementary Figs. 2 to 4.

### GRADE

We graded the certainty of the evidence for the comparison between DOAC and VKA on dementia risk in AF patients as very low, due to the very serious risk of bias and serious inconsistency for the nine included studies.

The assessment of each parameter of the GRADE tool can be consulted in Supplementary Table 4.

## Discussion

### Summary of evidence

This systematic review suggests that the use of DOAC therapy in AF patients is associated with a significant reduction in dementia risk when compared with VKA. This meaningful result was reassured by the most robust analysis of the nine studies including exclusively dementia as the outcome.

We have hypothesized that DOACs could be more protective than VKA because they showed a better efficacy and/or safety profile than warfarin [37, 38]. Additionally, continuous use of warfarin, as required in AF therapy, is associated with a significantly increased risk of bleeding and an increasing probability of lack of adherence to VKA therapy, which in turn contributes to an unstable INR and consequently to an increased risk of stroke [39]. This is a clear limitation against its use, when compared to DOACs, since this newer anticoagulation therapy is based on a more comfortable and predictable dose–response profile, having fewer interactions, faster onset of action, and no need for monitoring and adjusting the doses, as well as being associated with higher patient satisfaction [40], all of which are useful to increase patient adherence to therapy.

It is important to highlight that DOAC’s true beneficial effect on dementia risk might have been hindered due to DOAC’s tendency to be more often prescribed when treatment is initiated during hospitalization [41], thus including patients with more uncontrolled comorbidities and consequently more susceptible to neurocognitive impairment. Consequently, its effect may be higher than the results presented in this review. However, we observed an equilibrium of dementia risk factors between both interventions, which contradicts the above-mentioned premise. Yet, dementia’s risk factors are much more ubiquitous than the risk factors reported [42] and so it is hard to guarantee they were balanced among both groups. Furthermore, there is no data regarding the severity or management of included patients' comorbidities, both of which are important factors contributing to confounding beyond our control.

The included studies had follow-up periods ranging from 243 days to 9 years. According to current evidence, dementia is a slowly progressing disease with a long latency period from the best period for intervention to its occurrence [25]. Consequently, one might think the longer the follow-up period, the sturdier the results, since more events would be accounted for and a better understanding of the DOACs and VKAs' effect on dementia risk would be accomplished.

Nonetheless, our longer follow-up period subgroup did not show a significant reduction in dementia risk, probably because the longer follow-up period study included was only 9 years. Longer studies might assert more expressive results regarding this matter. However, long follow-up period studies would probably be associated with increased loss to follow-up and substantial costs, precluding large-scale studies. Furthermore, age is a strong risk factor for dementia [43], but the benefit of recruiting older patients would be attenuated by the high mortality rate (of around 13% in the population with ≥ 85 years old) [44], particularly in studies with more than 5 years. As such, modest follow-up periods (e.g. 4–5 years) are the best choice for analysing differences in dementia incidence, particularly in high risk populations.

That being said, high risk patients such as (1) patients with mild cognitive impairment (MCI), defined as a change in cognition, impairment in one or more cognitive domains and preservation of independence in functional abilities and social or occupational functioning (MCI NIA-AA criteria) [45], (2) patients with known biomarkers associated to cognitive impairment (eg. small vessels disease assessment through MRI or tau and/or amyloid protein assessment through PET scan) and (3) patients with a higher risk of inherit dementia (eg. familiar history of dementia and/or patients with APOE ε4 polymorphisms) might be beneficial to include in studies evaluating dementia risk.

However, there are some important aspects to mention: firstly, the clinical course of mild cognitive impairment (MCI) is not always predictable (patients can improve, remain stable or progress to dementia) [46]; secondly, risk biomarkers are expensive to assess in large scale, their causality to dementia is yet to be established, particularly in older ages, and in shorter studies biomarkers endpoints might miss beneficial effects of an intervention; and thirdly, the results obtained in studies including this higher risk population could not be extrapolated to sporadic dementia in the general population [25].

DOACs did not achieve statistically significant risk reduction in the composite outcome studies. Furthermore, the beneficial overall effect on the primary outcome did not persist in the remaining seven studies after the exclusion of the composite outcome subgroup, most likely due to the effect of the cardiovascular and mortality components on the subgroup's overall effect. Current evidence demonstrates that DOACs are superior to warfarin in preventing stroke and systemic embolism [47, 48] and result in lower mortality [48], and, as such, these components probably had a positive effect on the assessed outcome, influencing the real effect of DOACs’ intervention.

Current evidence states that AF is associated with a four to five-fold risk increment of ischemic stroke [18, 49] and silent brain infarction [50], both of which increase the risk of dementia [51, 52], regardless of the symptoms and durations of AF [53]. Cardioembolic events are thought to be of great clinical relevance as seen in the post-stroke and vascular dementia new-onset cases. Hence, having DOACs a better anticoagulation control as stated in the previous paragraph, one would expect a more favourable effect of DOACs, particularly in vascular dementia. However, due to the lack of data concerning the different types of dementia diagnosed, we could not make a vascular dementia subgroup analysis, hereby expressing the need for more data regarding this subject.

We obtained similar results to a previous systematic review by Lee et al. [54]. However, our study included a higher number of studies from large national databases with more patients and longer follow up periods, thus allowing a better understanding of the effects of anticoagulation in AF patients in a real-world setting. Furthermore, our study had stricter inclusion criteria for the meta-analysis, by only including patients with no previous diagnosis of dementia, thus avoiding biased results; and by excluding studies without the outcome of dementia, such as the included RCTs in said systematic review [47, 48, 55, 56].

Due to the current absence of randomized controlled studies regarding this subject, there is a need for more controlled studies to obtain sufficient quality evidence to draw definitive conclusions about which group of oral anticoagulants has a lower risk of dementia associated. Ideally, there should be conducted a double-blinded randomized control trial, including only patients with documented non-valvular atrial fibrillation and no previous diagnosis of dementia or mild cognitive impairment (MMSE > 25 or equivalent by other validated diagnostic tool), and no other indication for anticoagulation or antiplatelet therapy. The creation of subgroups according to the patients’ age (< 65 years old, 65–75 years old and > 75 years old) might help clarify the effects of anticoagulation therapy between different age groups. There should be two treatment arms: DOACs and VKA. Active substances, doses, TTR and posology should be documented and preferably homogenous among the participants of the study. Outcomes should include cognitive impairment, dementia, vascular dementia and Alzheimer’s dementia. Cognitive impairment and dementia diagnosis should be well documented and the follow-up period should be longer than 5 years. Secondary outcomes might include strokes, transient ischemic attacks (TIA) and intracranial bleeding.

Currently, there are four ongoing randomized controlled trials (Clinicaltrials.gov identifier: NCT02387229; NCT03061006; NCT01994265; UminClinicalTrials identifier: UMIN000025721) comparing the effects between DOAC and VKA therapy on AF patients regarding dementia risk.

### Strengths and limitations

The main strength of our systematic review is its major importance for today's society, as the analysis of such an issue can identify better therapies preventing dementia risk in AF patients and, therefore, have a considerable impact on millions of patients. Also of significance, our review included 1,175,609 patients from various national databases, hence creating a representable sample size of the population in the study.

On the other hand, our meta-analysis was based on observational studies and, as such, the data presented is prone to bias, in particular, selection bias, since DOACs could have been favoured over VKA in patients with suspected cognitive incapacity or anticipated difficulty in medication management, for being a more comfortable and predictable therapy, without the need of monitoring and adjusting doses. However, observational studies give a more accurate representation of the real world than RCTs, which only include a very selected sample of the general population.

Another important limitation of our study is the fact that dementia was assessed through different criteria (such as ICD-9/ICD-10, MMSE cut-offs, administrative codes, and others), hence creating a potential source of discrepancy between studies; furthermore, the differentiation of the pathological substrates of MCI (mild cognitive impairment) and dementia is important for clinical research, as clinical criteria to diagnose these entities are distinct according to this substrate. Yet, most studies (1) did not accurately differentiate these conditions, (2) used criteria not sensitive enough to diagnose some type of dementia (e.g. ICD-9/10) and (3) wrongly included some ICD-9/10 codes as dementia when said codes classify completely different pathologies from the outcome in the study, as evidenced in Supplementary table 6.

It is also important to note that there were included several studies without information regarding time in therapeutic range (TTR) of patients on warfarin treatment. Stricter criteria to included adequately anticoagulated patients would provide sturdier results. Nonetheless, the three studies that revealed said TTR values had most patients adequately coagulated.

Finally, there was significant heterogeneity of clinical characteristics and interventions across the different studies, such as the use of different DOACs, use of the same DOAC at different dosages, comorbidities, co-medications, and others. Of special significance, the data of Kundnani et al. [35] regarding the comparison of apixaban and acenocoumarol should be analysed with caution, since its extrapolation to a broader comparison between DOACs and VKAs may not reflect the true effects of said anticoagulant classes on dementia risk, but rather the individual effects of these particular drugs.

## Conclusion

In patients with AF, DOAC therapy was associated with a significant decrease in the risk of dementia when compared with VKA therapy. However, there is a need for higher quality studies, to better confirm the impact of DOAC therapy in AF patients regarding dementia outcomes, when compared with VKA therapy. Therefore, due to the very low certainty of the evidence and the paucity of clinical trials dedicated to answering this clinically important question underscores a need for global clinical research initiatives.

## Supplementary Information

Below is the link to the electronic supplementary material.

Supplementary file1 (DOCX 382 KB)

## Data Availability

The data evaluated were derived from published articles and the estimates are shown in the forest plots.
