# Supplementary material for: Direct oral anticoagulants vs vitamin K antagonist on dementia risk in atrial fibrillation: systematic review with meta-analysis
Source: J Thromb Thrombolysis. 2023 Jul 5;56(3):474–84. doi: 10.1007/s11239-023-02843-5 (PMC10439029; doi:10.1007/s11239-023-02843-5)
Supplement: Supplementary file 1 — Supplementary file1 (DOCX 382 KB) [file 11239_2023_2843_MOESM1_ESM.docx]

**Supplementary Material**

Effects of Non-Vitamin K Antagonist Oral Anticoagulants versus Vitamin K Antagonist Therapy on Dementia Risk in Patients with Atrial Fibrillation: Systematic Review with Meta-Analysis

Authors: Diogo R Branco, Mariana Alves, Catarina Severiano Sousa,

João Costa, Joaquim J Ferreira, Daniel Caldeira

**Corresponding author**

Professor Joaquim Ferreira

Laboratory of Clinical Pharmacology and Therapeutics, Faculdade de Medicina, Universidade de Lisboa, Portugal. Av. Prof. Egas Moniz, Lisboa 1649-028, Portugal; [joaquimjferreira@gmail.com](mailto:joaquimjferreira@gmail.com)

Contents

[Supplementary table 1: Search strategy 3](#_Toc95377968)

[Supplementary table 2: Primary outcomes and respective HRs 4](#_Toc95377969)

[Supplementary table 3: Risk of bias 5](#_Toc95377970)

[Supplementary table 4: PICOS 5](#_Toc95377971)

[Supplementary table 5: Dementia criteria 5](#_Toc95377972)

[ICD-10 codes 6](#_Toc95377973)

[Conversion table: ICD-9 / ICD-10 10](#_Toc95377974)

[Conversion table: ICD-8 / ICD-10 12](#_Toc95377975)

[Supplementary figure 1: Hazard ratio for risk of bias subgroup analysis (moderate vs serious risk) 14](#_Toc95377976)

[Supplementary figure 2: Hazard ratio for follow-up period subgroup analysis (short vs long follow-up) 14](#_Toc95377977)

[Supplementary figure 3: Hazard ratio for outcome subgroup analysis (single vs composite outcome) 15](#_Toc95377978)

[Supplementary figure 4: Egger Test 15](#_Toc95377979)

# **Supplementary table 1:** Search strategy

| **#** | **Searches** |
| --- | --- |
| 1 | atrial fibrillation.af. |
| 2 | (Atrial adj1 Fibrillat*).af. |
| 3 | (Auricular adj1 Fibrillat*).af. |
| 4 | atrial flutter.af. |
| 5 | auricular flutter.af. |
| 6 | Afib*.af. |
| 7 | a-fib*.af. |
| 8 | 1 or 2 or 3 or 4 or 5 or 6 or 7 |
| 9 | direct oral anticoagulant*.af. |
| 10 | Direct Oral Anti-coagulant*.af. |
| 11 | DTI*.af. |
| 12 | Factor Xa Inhibitor*.af. |
| 13 | Fxa inhibitor*.af. |
| 14 | ((new or novel or newer or non-vitamin K or nonvitamin K or target-specific or target specific) adj3 anticoagulant*).af. |
| 15 | rivaroxaban.af. |
| 16 | dabigatran.af. |
| 17 | edoxaban.af. |
| 18 | apixaban.af. |
| 19 | xarelto.af. |
| 20 | savaysa.af. |
| 21 | lixiana.af. |
| 22 | pradaxa.af. |
| 23 | eliquis.af. |
| 24 | anticoagulant*.ti,ab. |
| 25 | (Delirium or deliri*).af. |
| 26 | (Dementia or dement*).af. |
| 27 | (Cogniti* adj2 disorder*).af. |
| 28 | Memory disorder*.af. |
| 29 | Amnestic disorder* {Including Related Terms} |
| 30 | Alzheimer Disease {Including Related Terms} |
| 31 | (Dementia adj1 (senile or presenile)).af. |
| 32 | exp Dementia, vascular/ |
| 33 | exp dementia/ |
| 34 | Frontotemporal lobar degeneration.af. |
| 35 | Lewy body disease.af. |
| 36 | (Cognitive adj1 impairment*).af. |
| 37 | (Cognitive adj1 decline).af. |
| 38 | (Cognitive adj1 loss).af. |
| 39 | (Cognitive adj1 disorder).af. |
| 40 | (Cognitive adj1 dysfunction).af. |
| 41 | Neurocognitive disorder*.af. |
| 42 | Mild cognitive impairment.af. |
| 43 | exp Frontotemporal Dementia/ |
| 44 | exp Dementia, Multi-Infarct/ |
| 45 | exp Cognitive Dysfunction/ |
| 46 | Mini Mental State Examination.af. |
| 47 | mmse.af. |
| 48 | Montreal Cognitive Assessment {Including Related Terms} |
| 49 | moca.af. |
| 50 | COGNISTAT.af. |
| 51 | Mini-Cog.af. |
| 52 | "dementia rating".af. |
| 53 | microcog.af. |
| 54 | Cognitive Assessment Screening Instrument.af. |
| 55 | 25 or 26 or 27 or 28 or 29 or 30 or 31 or 32 or 33 or 34 or 35 or 36 or 37 or 38 or 39 or 40 or 41 or 42 or 43 or 44 or 45 or 46 or 47 or 48 or 49 or 50 or 51 or 52 or 53 or 54 |
| 56 | 9 or 10 or 11 or 12 or 13 or 14 or 15 or 16 or 17 or 18 or 19 or 20 or 21 or 22 or 23 OR 24 |
| 57 | 8 and 55 and 56 |
| 58 | remove duplicates from 58 |

# **Supplementary table 2:** Primary outcomes and respective HRs

| Study year | | Primary Outcome | HR |
| --- | --- | --- | --- |
| Chen et al. 2018 | | Dementia | HR 0.85, 95%CI (0.71; 1.01) |
| Friberg et al. 2019 | | Dementia | HR 0.97, 95%CI (0.67; 1.40) |
| Jacobs et al. 2016 | | Composite outcome of dementia, stroke, and TIA | HR 0.42, 95%CI (0.18; 0.96) |
| Kim et al. 2020 | | Dementia | HR 0.78, 95%CI (0.68; 0.90) |
| Mongkhon et al. 2020 | | Composite of new-onset dementia/cognitive impairment | HR 0.89, 95%CI (0.69; 1.14) |
| Sogaard et al. 2019 | 60 – 69 years old | Dementia | HR 0.92, 95%CI (0.48; 1.76) |
|  | 70 – 79 years old |  | HR 0.86, 95%CI (0.68; 1.09) |
|  | 80 or more years old |  | HR 1.31, 95%CI (1.07; 1.59) |
| Hsu et al. 2021 | | Dementia | HR 0.82, 95%CI (0.73; 0.92) |
| Kundnani et al. 2021 | | Dementia | HR 0.38, 95%CI (0.09; 1.63) |
| Lee et al. 2021 | | Dementia | HR 0.929, 95%CI (0.858; 1.007) |

# **Supplementary table 3:** Risk of bias

| Author | Bias due to confounding | Bias in the selection of participants into the study | Bias in classification of interventions | Bias due to deviations from intended interventions | Bias due to missing data | Bias in the measurements of outcome | Bias in the selection of reported result | **Overall risk of Bias** |
| --- | --- | --- | --- | --- | --- | --- | --- | --- |
| Chen et al. 2018 | Moderate | Moderate | Moderate | Low | Low | Moderate | Low | Moderate |
| Friberg et al. 2019 | Moderate | Low | Moderate | Low | Low | Serious | Low | Serious |
| Jacobs et al. 2016 | Moderate | Moderate | Moderate | Low | Low | Moderate | Low | Moderate |
| Kim et al. 2020 | Moderate | Low | Moderate | Low | Low | Moderate | Low | Moderate |
| Mongkhon et al. 2020 | Moderate | Low | Moderate | Low | Low | Serious | Low | Serious |
| Søgaard et al. 2019 | Moderate | Low | Moderate | Low | Low | Moderate | Low | Moderate |
| Hsu et al. 2021 | Moderate | Low | Moderate | Low | Low | Moderate | Low | Moderate |
| Kundnani et al. 2021 | Moderate | Low | Moderate | Low | Low | Moderate | Low | Moderate |
| Lee et al. 2021 | Moderate | Low | Moderate | Low | Low | Serious | Low | Serious |

# **Supplementary table 4:** PICOS

| Parameter | Inclusion criteria |
| --- | --- |
| Participants | Patients with atrial fibrillation who had no previous diagnosis of neurocognitive impairment or dementia. |
| Interventions | Oral anticoagulation defined as non-vitamin K antagonists / direct oral anticoagulants (apixaban, dabigatran, edoxaban, rivaroxaban). |
| Comparators | Oral anticoagulation defined as vitamin K antagonists (warfarin, phenprocoumon, acenocoumarol). |
| Outcomes | Incidence of dementia. |
| Study designs | Randomized controlled trials or observational studies. |

# **Supplementary table 5:** Dementia criteria

| **Study** | **MMSE (Mini Mental State Examination)** | **ICD-8** | **ICD-9** | **ICD-10** | |
| --- | --- | --- | --- | --- | --- |
|  |  |  |  | **Chapter V**  Mental and behavioral disorders  (F00-F99) | **Chapter VI**  Diseases of the nervous system  (G00-G99) |
| Friberg et al. 2019 (Ref 23) | NA | NA | NA | F00- Dementia in Alzheimer disease  F01- Vascular dementia  F02- Dementia in other diseases classified elsewhere  F03- Unspecified dementia | G051- **Encephalitis, myelitis and encephalomyelitis in viral diseases classified elsewhere**  G300- **Alzheimer disease with early onset**  G301- **Alzheimer disease with late onset**  G308- **Other Alzheimer disease**  G309- **Alzheimer disease, unspecified**  G310- **Other degenerative diseases of nervous system, not elsewhere classified- Circumscribed brain atrophy (**Frontotemporal dementia**, Pick disease, Progressive isolated aphasia)**  G311- **Other degenerative diseases of nervous system, not elsewhere classified- Senile degeneration of brain, not elsewhere classified (Alzheimer disease, Senility NOS)**  G312- **Other degenerative diseases of nervous system, not elsewhere classified- Degeneration of nervous system due to alcohol (Alcoholic cerebellar ataxia, alcoholic cerebellar degeneration, cerebral degeneration, encephalopathy, dysfunction of autonomic nervous system due to alcohol)**  G318- **Other degenerative diseases of nervous system, not elsewhere classified- Other specified degenerative diseases of nervous system (Grey-matter degeneration,** Lewy body dementia**/disease, subacute necrotizing encephalopathy)** |
| Jacobs et al. 2016 (Ref 24) | NA | NA | 290-294 (organic psychotic conditions)  **331 (other cerebral degenerations)**  (See ICD-9 code table) | NA | NA |
| Chen et al. 2018 (Ref 27) | NA | NA | 290 (dementia)  294 (persistent mental disorders attributable to conditions classified elsewhere)  **331** (Alzheimer disease)  (See ICD-9 code table) | NA | NA |
| Kim et al. 2020 (Ref 28) | NA | NA | NA | F00- Dementia in Alzheimer disease  F01- Vascular dementia  F02- Dementia in other diseases classified elsewhere  F03- Unspecified dementia, combined with use of one or more of dementia drugs (rivastigmine, galantamine, memantine, donepezil) | **G30**- Alzheimer disease (ver lista de códigos)  G31- Other degenerative diseases of nervous system, not elsewhere classified (See ICD-10 code table) |
| Mongkhon et al. 2020 (Ref 29) | NR* | NR* | NR* | NR* | NR* |
| Sogaard et al. 2019 (Ref 30) | NA | Alzheimer dementia:  290.10  290.09  Vascular dementia:  293.09  293.19  Other dementia:  **094.19**  290.11-290.19 | NA | Alzheimer dementia:  F00  Vascular dementia:  F01  Other dementia:  F02-F03  F10.73-F19.73 | Alzheimer dementia:  **G30**  Other dementia:  **G23.1**  G31.0A  **G31.0B**  **G31.1**  G31.8B  **G31.8E**  **G31.85** |
| Hsu et al. 2021 (Ref 31) | NA | NA | 290.0-290.4- Ver lista  **331.0-** Alzheimer disease | F01- Vascular dementia  F03- Unspecified dementia | **G30**- Alzheimer disease |
| Lee et al. 2021 | NR* | NR* | NR* | NR* | NR* |
| Kundnani 2021 | NR** | | | | |

NA: Not applicable

NR: Not reported

* Supplement data not available.

** The authors state that they used MMSE and SCL-90 for neuropsychiatric evaluations. They do not mention the exact criteria used.

## **ICD-10 codes**

| F00 Dementia in Alzheimer's disease |
| --- |
| F00.0*Dementia in Alzheimer disease with early onset |
| F00.1*Dementia in Alzheimer disease with late onset |
| F00.2*Dementia in Alzheimer disease, atypical or mixed type |
| F00.9*Dementia in Alzheimer disease, unspecified |
| F01 Vascular dementia |
| F01.0 Vascular dementia of acute onset |
| F01.1 Multi-infarct dementia |
| F01.2 Subcortical vascular dementia |
| F01.3 Mixed cortical and subcortical vascular dementia |
| F01.8 Other vascular dementia |
| F01.9 Vascular dementia, unspecified |
| F02 Dementia in other diseases classified elsewhere |
| F02.0 Dementia in Pick's disease |
| F02.1 Dementia in Creutzfeldt-Jakob disease |
| F02.2 Dementia in Huntington's disease |
| F02.3 Dementia in Parkinson's disease |
| F02.4 Dementia in human immunodeficiency virus [HIV] disease |
| F02.8 Dementia in other specified diseases classified elsewhere |
| F03 Unspecified dementia |
| F10 Mental and behavioral disorders due to use of alcohol |
| F10.0 Acute intoxication |
| F10.1 Harmful use |
| F10.2 Dependence syndrome |
| F10.3 Withdrawal state |
| F10.4 Withdrawal state with delirium |
| F10.5 Psychotic disorder |
| F10.6 Amnesic syndrome |
| F10.7 Residual and late-onset psychotic disorder |
| F10.8 Other mental and behavioral disorders |
| F10.9 Unspecified mental and behavioral disorder |
| F11 Mental and behavioral disorders due to use of opioids |
| F11.0 Acute intoxication |
| F11.1 Harmful use |
| F11.2 Dependence syndrome |
| F11.3 Withdrawal state |
| F11.4 Withdrawal state with delirium |
| F11.5 Psychotic disorder |
| F11.6 Amnesic syndrome |
| F11.7 Residual and late-onset psychotic disorder |
| F11.8 Other mental and behavioral disorders |
| F11.9 Unspecified mental and behavioral disorder |
| F12 Mental and behavioral disorders due to use of cannabinoids |
| F12.0 Acute intoxication |
| F12.1 Harmful use |
| F12.2 Dependence syndrome |
| F12.3 Withdrawal state |
| F12.4 Withdrawal state with delirium |
| F12.5 Psychotic disorder |
| F12.6 Amnesic syndrome |
| F12.7 Residual and late-onset psychotic disorder |
| F12.8 Other mental and behavioral disorders |
| F12.9 Unspecified mental and behavioral disorder |
| F13 Mental and behavioral disorders due to use of sedatives or hypnotics |
| F13.0 Acute intoxication |
| F13.1 Harmful use |
| F13.2 Dependence syndrome |
| F13.3 Withdrawal state |
| F13.4 Withdrawal state with delirium |
| F13.5 Psychotic disorder |
| F13.6 Amnesic syndrome |
| F13.7 Residual and late-onset psychotic disorder |
| F13.8 Other mental and behavioral disorders |
| F13.9 Unspecified mental and behavioral disorder |
| F14 Mental and behavioral disorders due to use of cocaine |
| F14.0 Acute intoxication |
| F14.1 Harmful use |
| F14.2 Dependence syndrome |
| F14.3 Withdrawal state |
| F14.4 Withdrawal state with delirium |
| F14.5 Psychotic disorder |
| F14.6 Amnesic syndrome |
| F14.7 Residual and late-onset psychotic disorder |
| F14.8 Other mental and behavioral disorders |
| F14.9 Unspecified mental and behavioral disorder |
| F15 Mental and behavioral disorders due to use of other stimulants, including caffeine |
| F15.0 Acute intoxication |
| F15.1 Harmful use |
| F15.2 Dependence syndrome  F15.3 Withdrawal state |
| F15.4 Withdrawal state with delirium |
| F15.5 Psychotic disorder |
| F15.6 Amnesic syndrome |
| F15.7 Residual and late-onset psychotic disorder |
| F15.8 Other mental and behavioral disorders |
| F15.9 Unspecified mental and behavioral disorder |
| F16 Mental and behavioral disorders due to use of hallucinogens |
| F16.0 Acute intoxication |
| F16.1 Harmful use |
| F16.2 Dependence syndrome |
| F16.3 Withdrawal state |
| F16.4 Withdrawal state with delirium |
| F16.5 Psychotic disorder |
| F16.6 Amnesic syndrome |
| F16.7 Residual and late-onset psychotic disorder |
| F16.8 Other mental and behavioral disorders |
| F16.9 Unspecified mental and behavioral disorder |
| F17 Mental and behavioral disorders due to use of tobacco |
| F17.0 Acute intoxication |
| F17.1 Harmful use |
| F17.2 Dependence syndrome |
| F17.3 Withdrawal state |
| F17.4 Withdrawal state with delirium |
| F17.5 Psychotic disorder |
| F17.6 Amnesic syndrome |
| F17.7 Residual and late-onset psychotic disorder |
| F17.8 Other mental and behavioral disorders |
| F17.9 Unspecified mental and behavioural disorder |
| F18 Mental and behavioral disorders due to use of volatile solvents |
| F18.0 Acute intoxication |
| F18.1 Harmful use |
| F18.2 Dependence syndrome |
| F18.3 Withdrawal state |
| F18.4 Withdrawal state with delirium  F18.5 Psychotic disorder |
| F18.6 Amnesic syndrome |
| F18.7 Residual and late-onset psychotic disorder |
| F18.8 Other mental and behavioral disorders |
| F18.9 Unspecified mental and behavioral disorder |
| F19 Mental and behavioral disorders due to multiple drug use and use of other psychoactive substances |
| F19.0 Acute intoxication |
| F19.1 Harmful use |
| F19.2 Dependence syndrome |
| F19.3 Withdrawal state |
| F19.4 Withdrawal state with delirium |
| F19.5 Psychotic disorder |
| F19.6 Amnesic syndrome |
| F19.7 Residual and late-onset psychotic disorder |
| F19.8 Other mental and behavioral disorders |
| F19.9 Unspecified mental and behavioral disorder |
| G05 Encephalitis, myelitis and encephalomyelitis in diseases classified elsewhere |
| G05.0 Encephalitis, myelitis and encephalomyelitis in bacterial diseases classified elsewhere |
| G05.1 Encephalitis, myelitis and encephalomyelitis in viral diseases classified elsewhere |
| G05.2 Encephalitis, myelitis and encephalomyelitis in other infectious and parasitic diseases classified elsewhere |
| G05.8 Encephalitis, myelitis and encephalomyelitis in other diseases classified elsewhere |
| G23 Other degenerative diseases of basal ganglia |
| G23.0 Hallervorden-Spatz disease |
| G23.1 Progressive supranuclear ophthalmoplegia [Steele-Richardson-Olszewski] |
| G23.2 Multiple system atrophy, parkinsonian type |
| G23.3Multiple system atrophy, cerebellar type |
| G23.8Other specified degenerative diseases of basal ganglia |
| G23.9Degenerative disease of basal ganglia, unspecified |
| G30 Alzheimer's disease |
| G30.0 Alzheimer's disease with early onset |
| G30.1 Alzheimer's disease with late onset |
| G30.8 Other Alzheimer's disease |
| G30.9 Alzheimer's disease, unspecified |
| G31 Other degenerative diseases of nervous system, not elsewhere classified |
| G31.0 Circumscribed brain atrophy (Frontotemporal dementia, Pick disease, Progressive isolated aphasia) |
| G31.1 Senile degeneration of brain, not elsewhere classified |
| G31.2 Degeneration of nervous system due to alcohol |
| G31.8 Other specified degenerative diseases of nervous system (Grey-matter degeneration, Lewy-body dementia/disease, Subacute necrotizing encephalopathy) |
| G31.9 Degenerative disease of nervous system, unspecified |

## **Conversion table: ICD-9 / ICD-10**

| **ICD-9** | **ICD-10** |
| --- | --- |
| 290 Senile and presenile organic psychotic conditions | No direct conversion |
| 290.0 Senile dementia, uncomplicated | F03.90 Unspecified dementia without behavioral disturbance |
| 290.1 Presenile dementia | No direct conversion |
| 290.10 Presenile dementia, uncomplicated | F03.90 Unspecified dementia without behavioral disturbance |
| 290.11 Presenile dementia with delirium | F03.90 Unspecified dementia without behavioral disturbance |
| 290.12 Presenile dementia with delusional | F03.90 Unspecified dementia without behavioral disturbance |
| 290.13 Presenile dementia with depressive | F03.90 Unspecified dementia without behavioral disturbance |
| 290.2 Senile dementia with delusional or depressive features | No direct conversion |
| 290.20 Senile dementia with delusional features | F03.90 Unspecified dementia without behavioral disturbance |
| 290.21 Senile dementia with depressive features | F03.90 Unspecified dementia without behavioral disturbance |
| 290.3 Senile dementia with delirium | F03.90 Unspecified dementia without behavioral disturbance |
| 290.4 Arteriosclerotic dementia | No direct conversion |
| 290.40 Arteriosclerotic dementia, uncomplicated | F01.50 Vascular dementia without behavioral disturbance |
| 290.41 Arteriosclerotic dementia with delirium | F01.51 Vascular dementia with behavioral disturbance |
| 290.42 Arteriosclerotic dementia with delusional features | F01.51 Vascular dementia with behavioral disturbance |
| 290.43 Arteriosclerotic dementia with depressive features | F01.51 Vascular dementia with behavioral disturbance |
| **290.8 Other specified senile psychotic conditions** | F03.90 Unspecified dementia without behavioral disturbance |
| **290.9 Unspecified senile psychotic condition** | F03.90 Unspecified dementia without behavioral disturbance |
| 291 Alcoholic psychoses | No direct conversion |
| **291.0 Alcohol withdrawal delirium** | **F10.231 Alcohol dependence with withdrawal delirium** |
| **291.1 Alcohol amnestic syndrome** | **F10.96 Alcohol use, unspecified with alcohol-induced persisting amnestic disorder** |
| 291.2 Other alcoholic dementia | F10.27 Alcohol dependence with alcohol-induced persisting dementia |
| **291.3 Alcohol withdrawal hallucinosis** | **F10.951 Alcohol use, unspecified with alcohol-induced psychotic disorder with hallucinations** |
| **291.4 Idiosyncratic alcohol intoxication** | **F10.929 Alcohol use, unspecified with intoxication, unspecified** |
| **291.5 Alcoholic jealousy** | **F10.950 Alcohol use, unspecified with alcohol-induced psychotic disorder with delusions** |
| **291.8 Other specified alcoholic psychosis** | No direct conversion |
| **291.9 Unspecified alcoholic psychosis** | **F10.99** **Alcohol use, unspecified with unspecified alcohol-induced disorder** |
| 292 Drug psychoses | No direct conversion |
| **292.0 Drug withdrawal syndrome** | **F19.939 Other psychoactive substance use, unspecified with withdrawal, unspecified** |
| **292.1 Paranoid and/or hallucinatory states induced by drugs** | No direct conversion |
| **292.11 Drug-induced organic delusional syndrome** | **F19.950 Other psychoactive substance use, unspecified with psychoactive substance-induced psychotic disorder with delusions** |
| **292.12 Drug-induced hallucinosis** | **F19.951 Other psychoactive substance use, unspecified with psychoactive substance-induced psychotic disorder with hallucinations** |
| **292.2 Pathological drug intoxication** | **F15.920 Other stimulant use, unspecified with intoxication, uncomplicated** |
| **292.8 Other specified drug-induced mental disorders** | No direct conversion |
| **292.81 Drug-induced delirium** | **F19.921 Other psychoactive substance use, unspecified with intoxication with delirium** |
| 292.82 Drug-induced dementia | F19.97 Other psychoactive substance use, unspecified with psychoactive substance-induced persisting dementia |
| **292.83 Drug-induced amnestic syndrome** | **F19.96 Other psychoactive substance use, unspecified with psychoactive substance-induced persisting amnestic disorder** |
| **292.84 Drug-induced organic affective syndrome** | **F19.94 Other psychoactive substance use, unspecified with psychoactive substance-induced mood disorder** |
| **292.89 Other** | F11-F19 (105 codes) |
| **292.9 Unspecified drug-induced mental disorder** | **F19.99 Other psychoactive substance use, unspecified with unspecified psychoactive substance-induced disorder** |
| **293 Transient organic psychotic conditions** | No direct conversion |
| **293.0 Acute delirium** | **F05 Delirium due to known physiological condition** |
| **293.1 Subacute delirium** | **F05 Delirium due to known physiological condition** |
| **293.8 Other specified transient organic mental disorders** | No direct conversion |
| **293.81 Organic delusional syndrome** | **F06.2 Psychotic disorder with delusions due to known physiological condition** |
| **293.82 Organic hallucinosis syndrome** | **F06.0 Psychotic disorder with hallucinations due to known physiological condition** |
| **293.83 Organic affective syndrome** | **F06.30 Mood disorder due to known physiological condition, unspecified** |
| **293.89 Other** | **F06.1 Catatonic disorder due to known physiological condition**  **F53 Puerperal psychosis** |
| **293.9 Unspecified transient organic mental disorder** | **F06.8 Other specified mental disorders due to known physiological condition** |
| 294 Other organic psychotic conditions (chronic) | No direct conversion |
| **294.0 Amnestic syndrome** | **F04 Amnestic disorder due to known physiological condition** |
| 294.1 Dementia in conditions classified elsewhere | No direct conversion |
| **294.8 Other specified organic brain syndromes (chronic)** | **F06.0 Psychotic disorder with hallucinations due to known physiological condition**  **F06.8 Other specified mental disorders due to known physiological condition** |
| **294.9 Unspecified organic brain syndrome (chronic)** | **F06.8 Other specified mental disorders due to known physiological condition** |
| **331 Other cerebral degenerations** | No direct conversion |
| **331.0 Alzheimer's disease** | **G30.9 Alzheimer disease, unspecified** |
| **331.1 Pick's disease** | No direct conversion |
| **331.2 Senile degeneration of brain** | **G31.1 Senile degeneration of brain, not elsewhere classified** |
| **331.3 Communicating hydrocephalus** | **G91.0 Communicating hydrocephalus** |
| **331.4 Obstructive hydrocephalus** | **G91.1 Obstructive hydrocephalus** |
| **331.5 Jakob-Creutzfeldt disease** | **G91.2 (Idiopathic) normal pressure hydrocephalus** |
| **331.6 Progressive multifocal leukoencephalopathy** | **G31.85 Corticobasal degeneration** |
| **331.7 Cerebral degeneration in other diseases classified elsewhere** | **G94 Other disorders of brain in diseases classified elsewhere** |
| **331.8 Other cerebral degeneration** | No direct conversion |
| **331.81 Reye's syndrome** | **G93.7 Reye's syndrome** |
| **331.89 Other** | **G31.89 Other specified degenerative diseases of nervous system** |
| **331.9 Cerebral degeneration, unspecified** | **G31.9 Degenerative disease of nervous system, unspecified** |

## **Conversion table: ICD-8 / ICD-10**

| **ICD-8** | **ICD-10** |
| --- | --- |
| **094 Syphilis of central nervous system** | No direct conversion |
| **094.0 Tabes dorsalis** | **A52.11 Tabes dorsalis** |
| **094.1 General paresis** | **A52.17 General paresis** |
| **094.9 Other syphilis of central nervous system** | **A52.3 Neurosyphilis, unspecified** |
| 290 Senile and presenile dementia | No direct conversion |
| 290.0 Senile dementia | F00.1* Dementia in Alzheimer disease, late onset  F00.2* Dementia in Alzheimer’s disease, atypical or mixed type |
| 290.1 Presenile dementia | F00.0* Dementia in Alzheimer’s disease, early onset  F02.1* Dementia in Creutzfeldt-Jakob disease  F02.0* Dementia in Pick disease |
| 293 Psychosis associated with other cerebral condition | No direct conversion |
| 293.0 Cerebral arteriosclerosis | F01.1 Multi-infarct dementia  F01.3 Mixed cortical and subcortical vascular dementia |
| 293.1 Other cerebrovascular disturbances | F01.0 Vascular dementia of acute onset  F01.2 Subcortical vascular dementia  F01.8 Other vascular dementia |
| **293.2 Epilepsy** | **Use 2 codes: one from the list of disorders mentioned for 292, plus an additional code to specify the associated condition:**  **G40 Epilepsy** |
| **293.3 Intercranial neoplasm** | **Use 2 codes: one from the list of disorders mentioned for 292, plus one of the following codes to specify the associated condition:**  **C70.—Malignant neoplasm of meninges**  **C71.—Malignant neoplasm of brain**  **D32.0 Benign neoplasm of cerebral meninges**  **D33.—Benign neoplasm of brain and other parts of central nervous system** |
| 293.4 Degenerative diseases of central nervous system | Use 2 codes: one from the list of disorders mentioned for 292, plus an additional code to specify the associated condition:  F02.2* Dementia in Huntington disease |
| **293.5 Brain trauma** | **Use 2 codes: one from the list of disorders mentioned for 292, plus an additional code to specify the associated brain trauma, such as:**  **S06.—Intracranial injury**  **S02.—Fracture of skull and facial bones**  **S07.—Crushing injury of head**  **P10.—Intracranial laceration and hemorrhage due to birth injury**  **P20.—Intrauterine hypoxia**  **P21.—Birth asphyxia** |
| 293.9 Other and unspecified cerebral condition | F02.8 Dementia in other diseases classified elsewhere  F03 Unspecified dementia  **F04 Organic amnesic syndrome, not induced by alcohol or other psychoactive substances.**  **F05.9 Unspecified delirium**  **F06.9 Unspecified mental disorder, due to brain damage and dysfunction and to physical disease**  **F09 Unspecified organic or symptomatic mental disorder** |


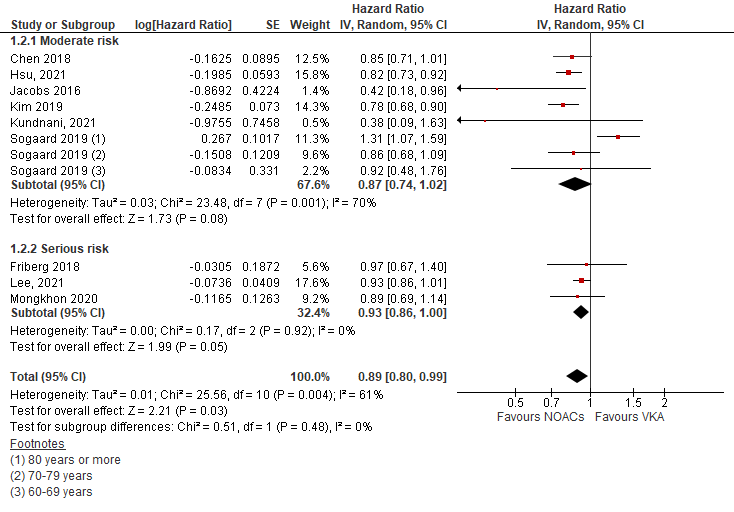


# **Supplementary figure 1:** Hazard ratio for risk of bias subgroup analysis (moderate vs serious risk)


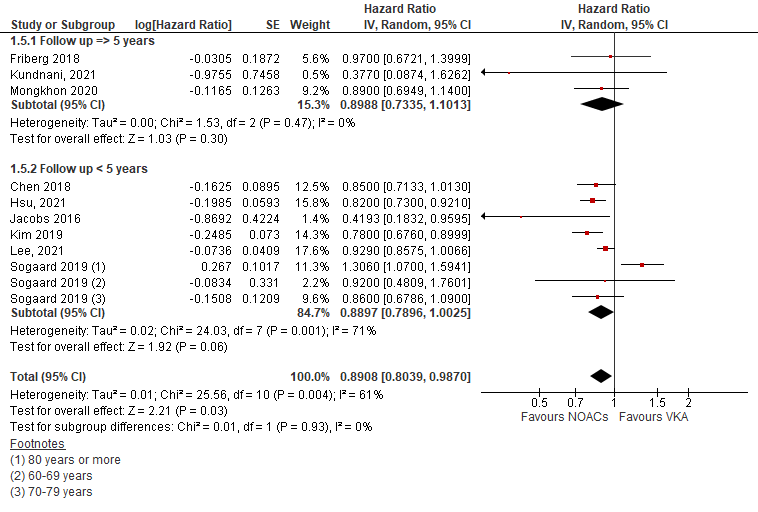


# **Supplementary figure 2:** Hazard ratio for follow-up period subgroup analysis (short vs long follow-up)


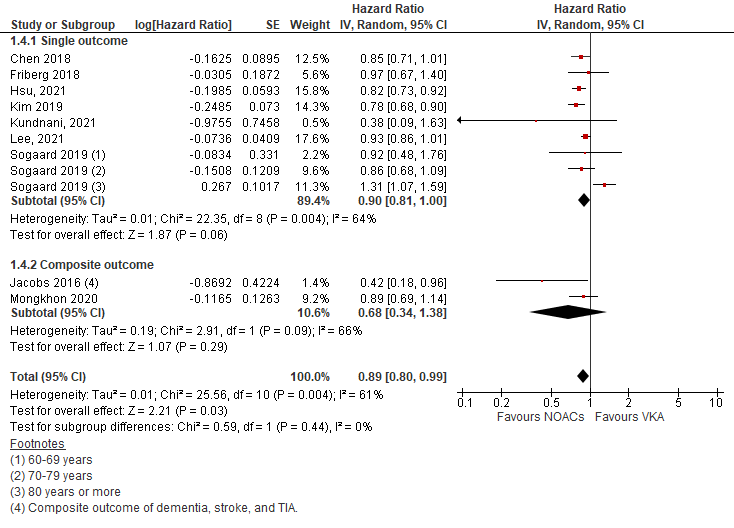


# **Supplementary figure 3:** Hazard ratio for outcome subgroup analysis (single vs composite outcome)

# **Supplementary figure 4:** Egger Test

No small-study effects / publication bias was detected (P = 0.586).
